# Survey and Comparative Study of LoRa-Enabled Simulators for Internet of Things and Wireless Sensor Networks

**DOI:** 10.3390/s22155546

**Published:** 2022-07-25

**Authors:** Sadiq Idris, Thenuka Karunathilake, Anna Förster

**Affiliations:** 1 Communication and Information Technology, University of Bremen, 28359 Bremen, Germany; 2 Sustainable Communication Networks, University of Bremen, 28359 Bremen, Germany; thenuka@comnets.uni-bremen.de (T.K.); anna.foerster@uni-bremen.de (A.F.)

**Keywords:** IoT, LoRa, LoRaWAN, LPWAN, simulation tools, WSN

## Abstract

The Internet of Things (IoT) is one of the most important emerging technologies, spanning a myriad of possible applications, especially with the increasing number and variety of connected devices. Several network simulation tools have been developed with widely varying focuses and used in many research fields. Thus, it is critical to simulate the work of such systems and applications before actual deployment. This paper explores the landscape of available IoT and wireless sensor networks (WSNs) simulators and compares their performance using the Low Power Wide Area Network (LPWAN) communication technology called LoRa (Long Range), which has recently gained a lot of interest. Using a systematic approach, we present a chronological survey of available IoT and WSNs simulation tools. With this, we categorized and content-analyzed published scientific papers in the IoT and WSNs simulation tools research domain by highlighting the simulation tools, study type, scope of study and performance measures of the studies. Next, we present an overview of LoRa/LoRaWAN technology by considering its architecture, transmission parameters, device classes and available simulation tools. Furthermore, we discussed three popular open-source simulation tools/frameworks, namely, NS-3, OMNeT++ (FLoRa) and LoRaSim, for the simulation of LoRa/LoRaWAN networks. Finally, we evaluate their performance in terms of Packet Delivery Ratio (PDR), CPU utilization, memory usage, execution time and the number of collisions.

## 1. Introduction

The recent rise of the Internet of Things (IoT)-connected devices is driving the increasing demand for advanced and new technologies. The IoT describes a vision in which billions of smart devices/things/objects are equipped with sensory and communication capabilities to autonomously sense, share and exchange information for intelligent decision making [1]. Such decisions can then be used in many applications such as agriculture, transportation, healthcare, climate change, supply chain management, etc. With little or no extensive infrastructure, wireless sensor networks (WSNs), a technology often used within an IoT system, play an important role in the IoT vision due to their robust design and self-organizing network concepts [2].

WSNs consist of several (hundreds or thousands) of low-power, low-cost tiny computers or sensor nodes deployed either randomly or in a predetermined manner in a given area of interest connected via wireless communication links [3,4,5,6,7]. They are specifically designed to sense some physical properties or conditions such as pressure, humidity, temperature, and vibration from their surrounding environment and send the collected data to at least a common gateway sensor node, called a sink or base station, via the internet in an IoT system [5,6,7].

Various communication technologies to interconnect IoT and WSNs devices have been developed. One such technology that has gained growing momentum and interest is the Low Power Wide Area Networks (LPWANs). They offer long-range, low-power consumption and wide-area coverage. Among the LPWAN technologies, four noticeable candidates, namely, Long Range (LoRa), Long-Term Evolution for Machines (LTE-M), Sigfox and Narrowband-IoT (NB-IoT), are showing the greatest acceptance. LoRa or LoRa Wide Area Network (LoRaWAN) technology has shown to be the most dominant of the four technologies in terms of the number of LoRaWAN network operators and the number of countries with established LoRaWAN networks [8]. It offers extended communication coverage, low-power consumption, low-cost, long battery life and high capacity potential [9,10].

Hence, this paper explores the landscape of available IoT and WSNs simulation tools and compares their performance using the LoRa communication technology. Our contributions are as follows:

- We present a chronological survey of available IoT and WSNs network simulators.
- We analyze and categorize recent studies between 2011 and mid-2021 with a focus on IoT and WSNs network simulation tools by highlighting the discussed simulators, study type, scope and performance measures of the studies.
- We examine and compare three popular open-source simulation tools/frameworks for the simulation of LoRaWAN networks in terms of packet delivery ratio (PDR), CPU utilization, memory usage, execution time and the number of collisions.

The rest of the paper is organized as follows. Section 2 provides an overview of the IoT architecture, review process and survey of available IoT and WSNs simulators. In Section 3, we exhibit an overview of the most popular LPWAN technologies, end device classes, transmission parameters and available simulation tools to analyze LoRa/LoRaWAN networks. Section 4 describes the methodological approach used in this work. In Section 5, we present our performance evaluation and results discussion. Finally, conclusions are drawn in Section 6.

## 2. Related Work

### 2.1. IoT: State-of-the-Art

Even though the IoT has no universally agreed-upon architecture, many researchers and industries have proposed various IoT architectures based on their own needs and requirements [11]. However, the three-layer architecture is the most generic or basic IoT architecture [12,13]. This architecture proposes three layers, namely, perception, network and application layer. The perception layer is the physical and main part of object identification and data collection [14]. It is sometimes called the sensing layer and has several sensor nodes, actuators and gateways that cooperatively sense, gather and exchange information about the environment. The network layer, also called the transmission layer, is responsible for transmitting and processing sensed data from the sensing layer to other network devices, servers and smart things/objects. This layer also handles all data transmission. On the other hand, the application layer is responsible for providing application-specific services to the end-user. This layer defines various IoT applications, such as smart agriculture, smart health systems, smart cities, etc. [11]. Moreover, many and different IoT architectures have been proposed in various literature, such as the four-layer [15], five-layer [16], and man-like neural network architecture [17].

### 2.2. Systematic Literature Review (SLR)

The SLR process used in this work is similar to that used in [18], and this is because it is well-suited for our purpose. The SLR protocol consists of four main steps:

- Search for the works in the domain of WSNs simulation tools: This step involves searching for published papers that discussed or mentioned WSNs simulation tools. The search was conducted on some of the most popular academic databases such as ACM, Elsevier, MDPI, Springer, IEEE Xplore and other digital libraries. In addition, the search used the following keywords: survey, comparison, review, simulator-specific, simulation tools, analytical studies, case studies, analytical study, qualitative analysis, technical report and evaluation with a focus on IoT and WSNs simulation tools. This step helps with retrieving and finding relevant papers from the pool of available scientific literature.
- Manually select the relevant papers: For this step, we manually select papers between 2011 and mid-2021, considering their relevance to the subject matter. All abstracts and conclusions sections were read to select the most relevant papers for the SLR process.
- Read and evaluate selected papers: For the third step, we carefully analyzed and examined the contents of the selected papers. This includes the year of publication, references, discussed or cited network simulators/emulators, type of study, scope and performance measures.
- Collect the most relevant data using the data extraction table: Finally, the most relevant data were collected using the data extraction table.

### 2.3. Categories of Selected Scientific Papers

Based on the type of study, we divided the selected papers into five groups:

**Group 1: Survey and Review papers.** The survey papers provide a general knowledge of WSNs simulators, such as features, advantages, disadvantages and classifications. These papers include [19,20,21,22,23,24,25,26,27,28,29,30,31,32,33,34,35,36,37,38,39,40,41,42,43,44,45,46,47,48,49,50,51]. In particular, the authors in [27,37] present a comprehensive survey of various simulation tools. In [27], the main features, advantages and disadvantages of four network simulators, namely, NS-2, J-Sim, NS-3 and OMNeT++, are discussed. The work presented in [37] describes 16 simulators, considering their features, limitations, methodology, test-beds and hardware platforms.

Review papers, on the other hand, describe WSN simulation tools in an in-depth or comprehensive way based on the available evidence. These papers include [52,53,54,55,56,57,58,59,60,61,62].

In [52], the authors present a review and comparison of 15 network simulators based on the type, network impairments, deployment mode and protocol support. They further proposed evaluation methodologies and techniques to help researchers choose the best simulation tool.

Reference [53] focused on the specifics of WSNs simulations, providing a state-of-art review, features and requirements of 11 well-known and used simulators suitable for WSNs simulations. The conclusion and recommendation drawn from the work are that WSN simulators require a proper energy model with harvesting simulation support, a model of the sensed environment, and a mobility framework with localization support. An in-depth overview of 24 simulation tools is presented in [54]. The work mainly focused on the components, features, structure, implementation and usage of the simulation tools.

Moreover, in [55], the seven most widely used simulation tools for WSNs based on a set of new preferred criteria, namely, scalability, accessibility, complexity, popularity, accuracy, models, protocols and extensibility are discussed. The work further identified key limitations of the simulators with emphasis on their suitability for simulating large-scale WSNs. In [56], researchers review 20 simulators and identify their features. Based on the usage, they classified them into three major domains of use: education, research, and industrial development and design.

The authors in [57] present statistical information on the seven most popular network simulators gathered during a literature survey of several research articles between 2000 and 2013. Following a simple comparison approach, they present an overview, main properties and background information on the popularity of the simulators. Based on their findings, they concluded that NS-3 and OMNeT++ simulators are good choices for academic researchers, with the latter option being better for researchers as it is more intuitive, easier to use and has a well-designed Graphical User Interface (GUI).

More than 30 simulation tools are described in [58], where their architecture, features, interface/GUI, and performance comparison are presented. The authors in [59] review 130 simulation environments for Ubiquitous Sensor Networks (USNs). The work further summarized the performance of several studies on simulation tools. Seven simulation tools are described and compared based on their license, sensor platform support, simulation code exportable, scalability, protocol design/optimization, mobile network simulation, dynamic network topology change, network support, standards, Medium Access Control (MAC) and routing support in [60].

Reference [61] examines 19 experimental tools and techniques for various WSNs applications selection based on their capabilities, ease of use, and accuracy. Finally, in [62], a comprehensive review of 12 simulation tools focusing on experimental analysis, modeling, estimation and avoiding interference is presented. The authors also provide insight into dealing with interference avoidance methods and improving coexistence mechanisms among various wireless devices operating in the same frequency band.

**Group 2: Comparison papers.** This group of papers includes comparisons and comparative studies of WSNs simulators based on defined criteria such as architecture, models, interface accessibility, user support, applications, extensibility, scalability, comparison tables, etc., to evaluate the differences between simulators. In addition, they also describe WSN simulators in a general way. These papers include [63,64,65,66,67,68,69,70,71,72,73,74,75,76,77,78,79,80,81,82,83,84,85,86,87].

Particularly, the authors in [63,74,78,81,82,83,87] perform a comparative analysis of various WSN simulation tools. In [63], the authors propose a comparative study of three simulation tools, namely, QualNet, OPNET and NS-2, using as reference a real testbed based on recent Imote2 sensors. In addition, they evaluate the impact of various MAC protocols with respect to the IEEE 802.15.4 standard. According to their findings, the NS-2 simulator gives the closest results to reality in the case of an indoor scenario, while in the case of an outdoor scenario, the OPNET simulator gives the best results.

A comprehensive study of 14 WSNs simulation tools is provided in [74]. Out of the 14 mainstream WSNs simulators discussed, the authors further performed a comparative study of six simulators (WSNet, Castalia, COOJA, MiXiM, PASES, and TOSSIM) by designing two simulation scenarios and comparing their performance based on the packet delivery ratio, network throughput, run-time performance, packet loss at the MAC layer, power consumption estimation accuracy and network latency. Their analysis shows that Castalia, COOJA and WSNet are very efficient for large-scale network modeling, while the computation resources and running time of MiXiM, TOSSIM, and PASES are large.

In [78], a survey and comparative study of 22 open-source WSNs is presented. The authors identified their characteristics and compared them in terms of energy consumption models, scalability, mobility model and extensibility. The authors in [81] compare 23 network simulators. They considered several perspectives, including features, supported protocols, components, simulation mode, platform, main applications, visual/visibility, accessibility, support, testing, advantages, disadvantages and limitations for their comparative analysis in multi-tables.

The work presented in [82] reviews the implementation and evaluation process in WSNs. They describe relevant testbeds, simulation tools and their features. Furthermore, they conducted an experimental study using these testbeds and simulations to highlight their pros and cons. They further implemented a localisation protocol as a used case to investigate the effectiveness of the Avrora and NS-2 simulators and two testbeds. Their work clarifies future work to improve the reliability, accuracy, and time consumption for better implementation.

In [83], the architecture, features, advantages and limitations of 10 network simulators are presented. The main objective of the study is to highlight the unique characteristics of a good simulator. In addition, the performance of MATLAB/Simulink, NS-2, NS-3 and OMNeT++ simulators using the Ad-hoc On-demand Distance Vector (AODV) routing protocol is evaluated.

Lastly, the authors in [87] present a study of 10 simulation tools for other ad hoc networks, such as Vehicular Ad hoc Network (VANET), WSN, Wireless Mesh Network (WMN), etc. They highlighted areas of strength, features, operating system, supported ad hoc technologies and degree of usability of the simulators.

**Group 3: Evaluation papers.** This group of papers focused on evaluating WSN simulation tools. These papers include [88,89,90,91]. The authors in [88] present evaluation approaches and requirements for sensor networks to enable credible, realistic and convenient WSN evaluation. They also compared simulation models and real-world wireless link behavior in various settings. In [89], an energy-aware model for WSNs is proposed. The proposed scheme ensures an energy consumption gain that considers time constraints. In [90], CupCarbon, a new WSN simulator, is presented. To evaluate the ease of use of the simulator, the authors proposed a modified version of the Dijkstra algorithm that includes the battery level of the nodes as an additional parameter for calculating the best route. The authors in [91] proposed a methodological approach to evaluate WSN simulators. Using this approach, they evaluated three WSNs simulators (NS-2, OMNeT++ and TOSSIM).

**Group 4: Case study papers.** This group of papers focused on exploring real-life contemporary or multiple WSNs systems. In [92], the authors evaluate five WSN simulation tools, namely, Castalia, MiXiM, PASES, WSNet and COOJA, using AODV protocol as a case study. They designed a multi-hop simulation scenario in each simulator and compared their performance. Despite the simulation analysis differences and the available component models, their results show the correctness of the benchmark methods adopted and proved the functional equivalence of the tools and their network model application for multi-hop. Reference [93] performs a quantitative and comparative analysis of six network simulators used for academic purposes. The study’s main objective is to identify the tools used to solve specific engineering problems in teaching–learning processes. The authors highlighted the importance of using different simulation tools, especially at the university and research environment, to promote scientific and/or technological solutions.

**Group 5: Analytical study and qualitative analysis papers.** This group of papers includes analytical studies and qualitative analysis. The authors in [94] present an analytical study of various network simulation tools and platforms focusing on associated main features. The study explored evaluation criteria, type of simulation, classification/categorization, designed or modified, nearby realistic experimental results and future directions. In [95], a qualitative analysis of 15 simulators for WSNs is presented. The authors also provide a detailed study and background of various WSN simulators, key features and limitations. Moreover, they compare the simulators in terms of type, event, license type, general or specific simulator, GUI support, pros and cons.

Table 1 presents a chronological overview contribution of the selected papers (2011–2021). Furthermore, Table 2 summarizes the comparative studies in [64,70,72,82,83,91,92,96,97] where the authors analyzed different performance measures such as delivery ratio, computational/execution time, memory usage, CPU utilization, delay, received packets, energy consumption, among others by simulating various test scenarios.

### 2.4. Statistical Analysis of Selected Papers

**Figure 1:**
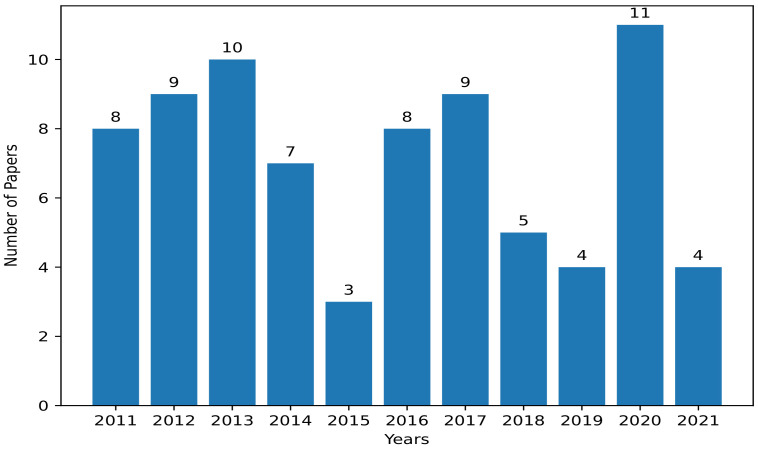
Yearly distribution of selected papers.

**Figure 2:**
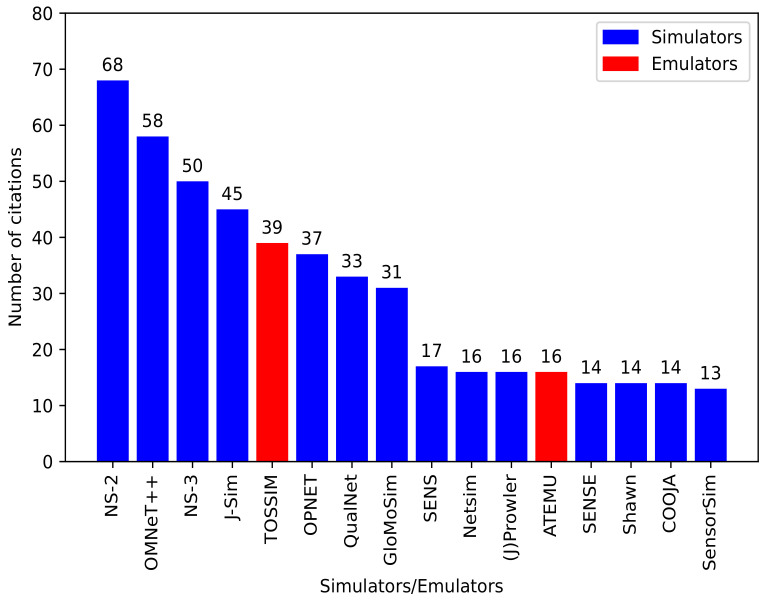
Number of WSN simulators/emulators citations.

In total, 78 relevant papers were obtained between 2011 and mid-2021. Group 1 has a total of 44 papers, which represent 56.4% of the selected papers. Group 2 has a total of 26 papers, which means 33.3% of the papers. Lastly, Groups 3, 4 and 5 have 4, 2, and 2 papers, representing 5.1%, 2.6% and 2.6% of the total selected papers, respectively. Moreover, Figure 1 shows the yearly distribution of the selected research papers. The year 2020 has the most obtained papers with a total of 11, followed by 2013 with 10, and 2012 and 2017 have 9 papers each. Even though many available simulators exist, as can be seen from Table 1, some of these simulators have higher citations than others. Figure 2 depicts the most cited simulators (14 simulators and 2 emulators) based on our analysis of the selected papers. Those simulators are, namely, NS-2, OMNeT++, NS-3, J-Sim, TOSSIM, OPNET, QualNet, GloMoSim, SENS, Netsim, (J)Prowler, ATEMU, SENSE, Shawn, COOJA, and SensorSim.

## 3. Low Power Wide Area Networks (LPWANs) Technologies

**Figure 3:**
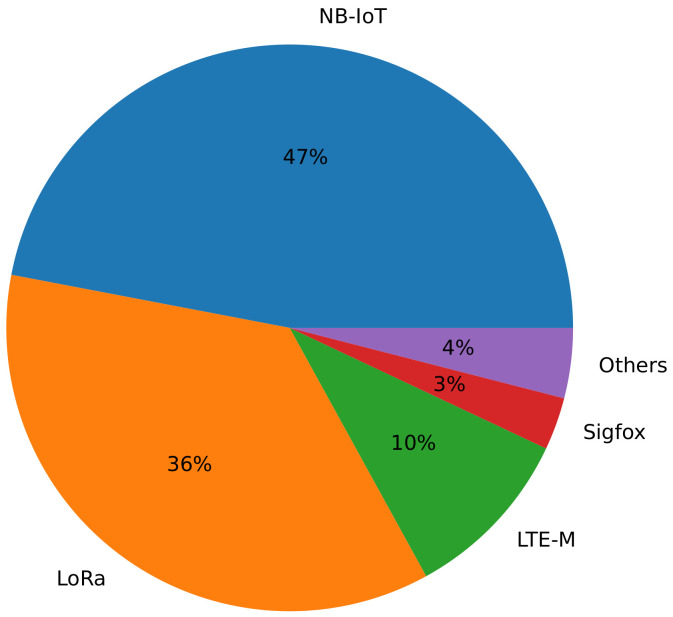
Technological distribution of installed LPWANs technologies base in 2021.

Today, LPWANs are becoming popular as a promising mechanism to connect billions of low-cost IoT devices. They are commonly used in many applications including smart environments [98], agriculture [99], environment monitoring [100], smart cities [101], and many more. Several LPWAN technologies are already present in the market, with Narrowband IoT (NB-IoT), LoRa/LoRaWAN, Sigfox and Long Term Evolution for Machines (LTE-M) accounting for over 96% of the global installed or deployed base of LPWAN-enabled active devices according to the market research conducted by IoT Analytics in 2021 [102]. According to their estimates, NB-IoT and LoRa lead with 47% and 36% (see Figure 3) of the global installed base, respectively.

Unlike NB-IoT and SigFox, LoRa/LoRaWAN allows for private network deployments and easy integration with various network platforms [103]. Since its introduction to the market, LoRaWAN has drawn the interest of many research communities and companies due to its unique features. In short, each LPWAN technology has distinct advantages over the others, especially considering various IoT factors. A comparison between LoRaWAN, NB-IoT, Sigfox, and LTE-M technologies can be found in [103,104,105].

### 3.1. Long Range (LoRa)

**Figure 4:**
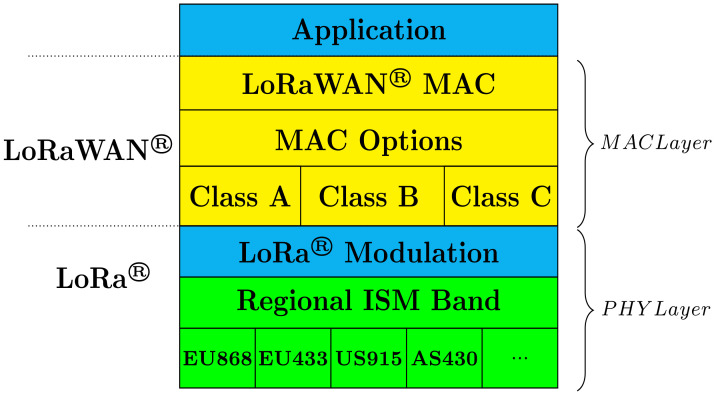
LoRaWAN protocol stack.

LoRa is a radio modulation technology in the category of LPWANs technologies used for IoT devices and applications [106,107,108,109,110,111,112,113,114,115,116,117]. It was first developed by a French company called Cycleo and later acquired in 2012 by Semtech Corporation [118]. Although LoRa and LoRaWAN are often used synonymously in the literature, they refer to two different concepts in the network. LoRa deals with only the physical (PHY) layer of the stack (see Figure 4), precisely, the wireless modulation used to utilize the long-range communication link. LoRaWAN, on the other hand, is the MAC layer protocol that acts mainly as an open networking protocol and is responsible for delivering secure bi-directional communication, localization services, security and mobility between LoRaWAN gateways and end-node devices [119,120]. Essentially, LoRaWAN enables IoT devices to communicate using the LoRa wireless technology. LoRaWAN is designed and maintained by the LoRa Alliance, which is an open, non-profit association of many companies and research institutions responsible for developing and standardizing the LoRAWAN specification.

Moreover, LoRa uses the Chirp Spread Spectrum (CSS) modulation technique, where information is carried using chirp signal [121]. A chirp is a signal whose frequency increases (up-chirp) or decreases (down-chirp) over time. LoRa operates in the unlicensed sub-GHz ISM (Industry, Science and Medical) radio frequency band that vary from country to country [121,122]. Table 3 shows the various unlicensed frequency bands and channel plans available for a given country or region. For example, the LoRaWAN networks in Europe are expected to operate between 863 and 870 MHz.

Furthermore, LoRaWAN has official regional parameters that can be found on the LoRa Alliance website [123], where various attributes of LoRaWAN link layer protocol specifications for different regions or regulatory environments worldwide are defined. These regional parameters specifications, which are maintained and provided by the LoRa Alliance, are aimed at assisting implementers in identifying the relevant LoRaWAN frequency bands and channel plans available by country. They include physical layer parameters such as channel frequencies, channel plans, join-request messages, data rates, and maximum payload size [123]. An overview of LoRa-Alliance regional parameters can be found in [124].

Currently, LoRa devices are used in various IoT applications to address some of the world’s biggest challenges ranging from smart cities [125], transportation [126], energy management [127], health monitoring [128], pollution control [129] and smart farming [130].

Moreover, three classes of end-devices, namely, Class A, B, and C, are defined in the LoRaWAN specification. Class A is the mandatory class for all LoRaWAN devices and is considered when end-devices (EDs) send data to the gateway at any time using ALOHA-based LoRaWAN MAC protocol [121]. Class B and C are extensions to Class A devices specification. In contrast to the other two classes, Class A is the most energy-efficient end-service system. Table 4 summarizes the main features and common applications of these classes.

**Figure 5:**
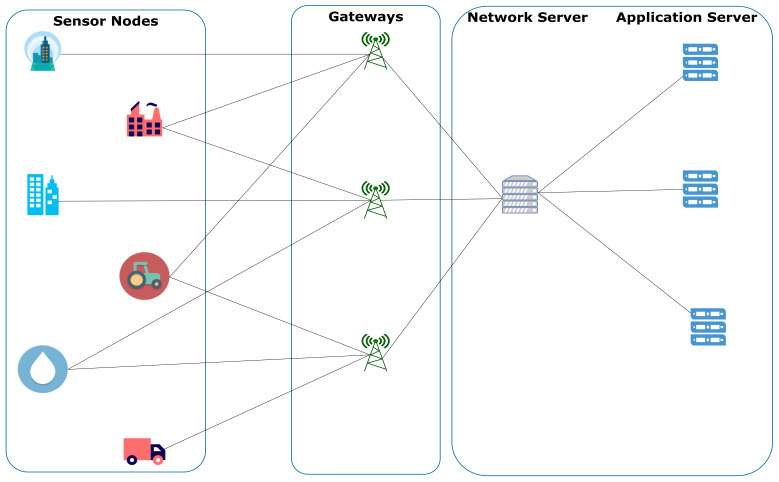
A typical LoRaWAN network architecture.

A typical LoRaWAN network architecture (see Figure 5) consists of four parts: LoRa end devices (EDs) or nodes, LoRa gateways, a network and an application server. The end nodes are LoRa devices with the LoRa radio modulation capability that run on powered batteries for several years. Typically, the EDs have embedded sensors, transponders and microcontrollers and are connected to the LoRa gateways using a star network topology. This is because long-range star architecture better preserves the battery lifetime [120]. After receiving LoRaWAN data from several LoRa nodes, the LoRa gateways channel the data to a network server and then to various application servers for end-user usage.

Furthermore, the communication between the nodes and the gateways is bi-directional, allowing the nodes to perform actuations. In addition, each node can transmit to multiple gateways. At the network server level, duplicate packets are automatically filtered out, and the appropriate data are forwarded to the correct application server. LoRaWAN technology is currently used in several IoT systems for solving many unlicensed wireless connectivity [133,134,135,136].

### 3.2. LoRa Transmission Parameters

Five configuration parameters, namely, Transmission Power (TP), Spreading Factor (SF), Bandwidth (BW), Coding Rate (CR) and Carrier Frequency (CF), characterize the communication between the LoRa EDs and LoRa gateway(s).

#### 3.2.1. Transmission Power (TP)

The TP is the power with which the transmitter sends a signal. The LoRa radio TP ranges from −4 to 20 dBm with 1 dB steps [137]. However, due to hardware implementation constraints, this range is often limited to 2 to 20 dBm [138]. The lower the TP value is, the longer the battery lifetime. Consequently, a lower TP value can decrease the transmission range. Moreover, the TP value for a particular frequency band is also a regional-dependent parameter. For example, the typical maximum transmit power for EU868-870, KR920-923 and IN865-867 is +16 dBm EIRP (+14 dBm ERP), +10 dBm EIRP (or +14 dBm EIRP) and +30 dBm EIRP, respectively. However, it is important to note that such TPs cannot be exploited whenever the LoRaWAN standard is adopted, while they are appropriate for LoRa modulation.

#### 3.2.2. Spreading Factor (SF)

The SF describes how the chirps would be spread out, i.e., the number of chirps generated by each symbol (chips/symbol) [139]. Its values range from 7 to 12. An SF of 8 (SF8) denotes that each chirp represents 8 bits. Higher SF values increase the Signal-to-Noise Ratio (SNR), network range, radio sensitivity and robustness against interference. However, the energy consumption and the packet airtime increase in this case [140]. On the other hand, a lower SF increases the payload, capacity and Time-on-Air (ToA) but decreases the transmission range by lowering the processing gain.

Moreover, because of its significant importance, the network uses SFs to control congestion. The SFs used by LoRa modulation are orthogonal; i.e., multiple spread signals can be transmitted on the same frequency channel simultaneously. Table 5 summarizes the effect of SF on the data rate, receiver sensitivity, battery life and ToA. The number of chips per symbol is calculated as 2^SF^. With an SF10, 1024 chips/symbol are used. However, such SFs, i.e., from 7 to 12, are the ones related to LoRaWAN, while when only LoRa transmission is adopted, the values of SFs can be selected between 6 and 12 [140]. With this, the spreading rate ranges between 2^6^ and 2^12^ chips/symbol. The relationship between SF, BW and chirp duration *(T_s_)* is given by [141]:

(1) $$2^{\mathrm{SF}}=\mathrm{BW}\cdot T_{s}$$

The modulation bit rate (*R_b_*) depends on the SF and is given by the relation [141]:

(2) $$R_{b}=\mathrm{SF}\cdot \frac{1}{\left[\frac{2^{\mathrm{SF}}}{\mathrm{BW}}\right]}=\mathrm{SF}\cdot \frac{\mathrm{BW}}{2^{\mathrm{SF}}}\left[\mathrm{bits}/\sec\right]$$

The symbol rate (*R_s_*) is the reciprocal of the *T_s_* expressed as:

(3) $$R_{s}=\frac{1}{T_{s}}=\frac{\mathrm{BW}}{2^{\mathrm{SF}}}\left[\mathrm{symbols}/\sec\right]$$

#### 3.2.3. Coding Rate (CR)

The CR refers to the LoRa modem’s forward error correction (FEC) rate that provides security/protection against interference [138]. The CR can be calculated as $\frac{4}{4+n}$ where *n*∈{1,2,3,4}. By substituting the values of *n*, the possible CR are 4/5, 4/6, 4/7 and 4/8. A CR of 4/5 (CR4/5) means that one bit of correction code will be added with every four bits of data. When CR = 0, no FEC is applied. A higher CR offers more protection against bursts of interference but increases the ToA and power consumption. LoRa radios with different CR settings can communicate with each other using an explicit header. This is because the CR payload stored in the header of the LoRa frame structure is always encoded at CR4/8 [142]. The nominal bit rate (*R_b_*) of the data signal can also be expressed in terms of the CR and BW as [141]:

(4) $$R_{b}=\mathrm{SF}\cdot \frac{\left[\frac{4}{4+\mathrm{CR}}\right]}{\left[\frac{2^{\mathrm{SF}}}{\mathrm{BW}}\right]}\left[\mathrm{bits}/\sec\right]$$

where SF ∈ {7,…,12} and CR ∈ {1,…,4} and rate code can be defined as $\frac{4}{4+\mathrm{CR}}$. Using Equation (4), the different nominal data rates computed with 125, 250 and 500 kHz are shown in Table 6. Clearly, a lower SF (for example, SF7) provides a higher bit rate than a higher SF (for example, SF12).

#### 3.2.4. Carrier Frequency (CF)

The CF refers to the central frequency between 137 and 1020 MHz (with steps of 61 Hz). This range may be limited to 860 to 1020 MHz depending on the LoRa chip and region. For example, the LoRaWAN protocol in Europe uses eight uplink channels defined inside the EU863-870 MHz free ISM band [143]. The Uplink and downlink channels can be used interchangeably on the first receiving window. Furthermore, a ninth uplink and downlink channel are defined at 868.8 MHz and 869.525 MHz, respectively. The ninth uplink channel uses the Frequency-Shift Keying (FSK) modulation, while the ninth downlink channel is only used for the second receiving window [143].

#### 3.2.5. Bandwidth (BW)

The BW describes the frequencies transmission band ranges over which LoRa’s chirps are spread. BW is one of the main parameters of the LoRa modulation and determines the chip rate of transmission according to Equation (1). A chip rate of 125 kcps corresponds to a bandwidth of 125 kHz. The LoRa network usually operates at either 125 kHz, 250 kHz or 500 kHz. The higher the BW, the higher the data rate, but the lower the radio sensitivity. In contrast, a lower BW results in higher radio sensitivity and lower data rate. Table 7 shows the possible bit rate and the maximum application payload size for the EU863-780 MHz ISM Band. The table shows that higher SF values decrease the bit rates, and lower SF values increase bit rates. However, for the same SF, doubling the BW also causes the data rate to double.

**Figure 6:**
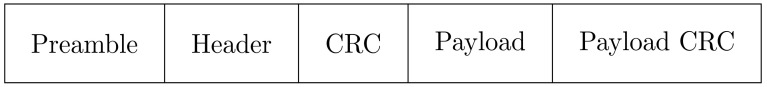
LoRa frame structure.

**Figure 7:**
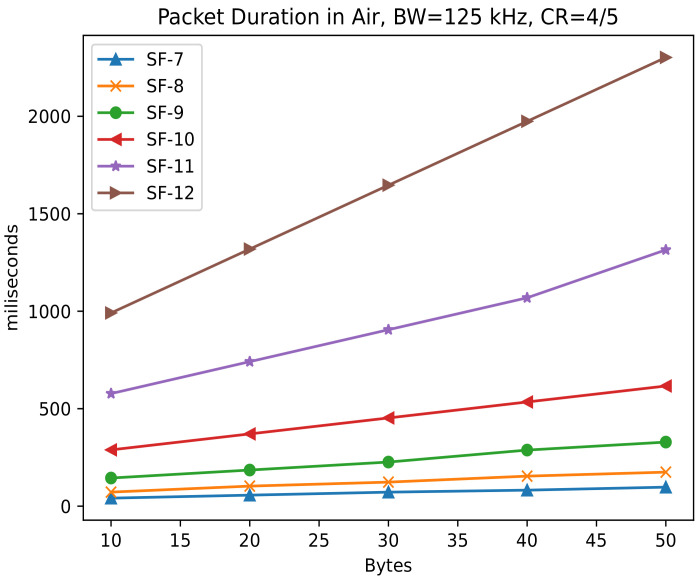
LoRa packet duration in air comparison.

Moreover, parameters such as the ToA and payload size of a packet can be derived from the previous parameters. Figure 6 shows the LoRa packet structure. The header in the structure can be either implicit or explicit. In most cases, the CR and Cyclic Redundancy Check (CRC) are known (enabled by default) and do not change, i.e., do not need to be specified (implicit header mode) [144]. The transmission time of a PHY layer packet or ToA can be calculated using Equations (5)–(8) as follows [144]:

(5) $$\mathrm{ToA}=T_{\mathrm{preamble}}+T_{\mathrm{payload}}$$

where *T_preamble_* is the preamble duration given by Equation (6) and *T_payload_* is the time to transmit payload given by Equation (7).

(6) $$T_{\mathrm{preamble}}=\left(n_{\mathrm{preamble}}+4.25\right)\cdot T_{\mathrm{sym}}$$

where *n_preamble_* is the programmed preamble length and *T_sym_*= $\frac{2\mathrm{SF}}{\mathrm{BW}}$ is the transmission time for one symbol.

(7) $$T_{\mathrm{payload}}=N_{\mathrm{payload}}\cdot T_{\mathrm{sym}}$$

where *N_payload_* is the number of payload symbols expressed as

(8) $$N_{\mathrm{payload}}=8+max\left(ceil\left[\frac{8\mathrm{PL}-4\mathrm{SF}+28+16\mathrm{CRC}-20\mathrm{IH}}{4(\mathrm{SF}-2\mathrm{DE}\;)}\right]\left(\mathrm{CR}+4\right),0\right)$$

where PL is the packet length in bytes, SF is the spreading factor, CRC is the cyclic redundancy check used for error detection of the LoRaWAN packet (CRC = 1 if enabled, 0 otherwise) and IH is the Implicit Header (0 if enabled, 1 otherwise). The DE value is set to 1 when the low data rate optimization is enabled; otherwise, it is disabled (DE = 0). Figure 7 shows the plot of the packet duration in air with varying payload from 10 to 50 bytes, BW = 125 kHz, CR = 4/5, *n_preamble_* = 8, IH = 0 and DE = 0.

### 3.3. An Overview of LoRa/LoRaWAN Simulation Tools

Simulation is undoubtedly essential for designing and evaluating of LoRa/LoRaWAN-based applications and networks before real deployment. Over the years, several LoRaWAN simulation tools have been developed by researchers for examining different LoRa applications and scenarios. While some are based on discrete events, others are developed specifically for LoRa/LoRaWAN networks. An overview of commonly used open-source simulation tools with a LoRa/LoRaWAN focus is presented in [145,146,147,148]. The most widely used simulation tools are LoRaSim, NS-3, OMNeT++ (FLoRa), CupCarbon, PhySimulator, SimpleIoTSimulator and Mbed OS Simulator. Table 8 compares LoRa/LoRaWAN simulators for IoT in terms of programming language, target domain (network generic or LoRa/LoRaWAN specific), operating system and available GUI.

Specifically, for this work, we will examine in detail the simulation tools that support the LoRa/LoRaWAN framework for carrying out LoRa/LoRaWAN network simulations. With this in mind, we have chosen NS-3, OMNeT++ (FLoRa) and LoRaSim for our analysis. The reasons for the selection is discussed in Step 1 (Section 4).

#### 3.3.1. LoRaSim

LoRaSim is a python-based discrete-event simulator designed to analyze the scalability of a LoRa network [137]. LoRaSim allows the deployment of *N* LoRa nodes (EDs) and *M* LoRa sinks (LoRa gateways or base stations) in a two-dimensional grid or random space. The channel model in LoRaSim is implemented based on the well-known log-distance path loss. Although LoRaSim is a simple simulator that provides great insights in terms of the network performance, however, acknowledgements (ACK) are not implemented [150]. Thus, it cannot be used to investigate the different aspects of network performance, especially when the nodes switch their SF based on the presence or absence of feedback from the gateway [150]. Moreover, LoRaSim only supports uplink transmissions and cannot be used to evaluate the Adaptive Data Rate (ADR) mechanism, which is essential for optimizing the network performance. It is worth mentioning that LoRaSim offers the possibility to run networks with multiple gateways by adjusting the SF and transmit power of the end node based on its distance from the gateway. For LoRaSim to work smoothly, packages such as SimPy, matplotlib and NumPy are required. It also offers a visualization plot of the network deployments but no graphical interface. Users can see much simulation information on the Command-Line Interface (CLI). LoRaSim has proved to be a big success in many research works. Many researchers have extended or improved it to suit their needs [156,157,161,163].

#### 3.3.2. Framework for LoRa (FLoRa)

FLoRa is a simulation framework that utilizes the OMNeT++ simulator and the INET framework for carrying out end-to-end simulations for LoRa networks [153]. It allows complete simulation of the LoRa/LoRaWAN network with its main components. FLoRa is implemented based on the LoRaWAN specification for class A EDs with unconfirmed transmission mode. Through the ADR mechanism, the network server and nodes support the dynamic management of configuration parameters [153]. The ADR mechanism controls the SF, BW and TP parameters of EDs. In contrast to other simulators, FLoRa provides a friendly user interface and a graphical representation of the network scenarios.

Moreover, FLoRa offers an accurate LoRa physical layer model and an end-to-end simulation with one (or more) gateways. The communication between the gateway(s) and the network server(s) is via the Internet Protocol (IP). The physical layer between the gateway(s) and the network server can be realized with the existing INET framework modules. However, FLoRa has its limitations and drawbacks. For example, it does not take into account any interference and mobility. Moreover, the ADR algorithm implemented in FLoRa does not support unconfirmed transmission mode, and the network server’s assigning of SFs is also not supported. To address some of the aforementioned problems, researchers in [165] have proposed a new simulator called Advanced Framework for LoRa (AFLoRa) based on the FLoRa simulator. AFLoRa is an updated version of the original FLoRa simulator with significant enhancements and additional LoRaWAN features. Many researchers have also validated their work using the FLoRa framework [166,167,168,169,170,171].

#### 3.3.3. LoRaWAN Module for NS-3

NS-3 is an open-source discrete-event network simulator designed primarily for educational and research purposes [172]. It is an extensible network simulation platform used under the GNU GPLv2 license. One of the fundamental design goals of NS-3 was to improve the realism of the models by allowing the model’s implementation closer to the actual software or real-world implementations that they represent. The core and models of NS-3 are implemented in the C++ programming language, with an optional Python Scripting API interface. Users can either use C++ *main()* or Python program to write their simulation scripts.

The LoRaWAN module for NS-3 is an extension of the NS-3 module for the simulation of LoRaWAN networks. Each LoRa end device and gateway of the LoRaWAN module for NS-3 contain a single LoRaWAN MAC/PHY pair component, and the interaction/communication between each end device’s PHY layer with its respective gateway’s PHY layer is through the spectrum channel module [151]. It supports LoRaWAN Class A EDs specifications. Moreover, the capture effect is the basis for the collision model used in the NS-3 LoRaWAN module. This effect occurs when two simultaneous uplink transmissions with the same frequency and SF collide, and the stronger signal captures the weaker signal. As a result, the gateway only receives the frame with the strongest received signal power. Many researchers over the years have developed different versions of NS-3 modules for the simulation of LoRaWAN networks. For the first time, the authors in [173] present a comprehensive survey of four different implementations of LoRaWAN modules in the NS-3 simulator. They labeled them as Module I through IV based on the date they were made publicly available and further compared them to highlight the most appropriate scenarios for each module. The four modules are available and free to download at GitHub, an internet code hosting platform for software development and version control. Most of the LoRaWAN specifications not found in the FLoRa framework are implemented in the NS-3 LoRaWAN module. In addition, compared to NS-3 LoRaWAN, FLoRa implementation is more difficult. Many researchers have validated, improved or extended their work using either the different implementations of the NS-3-based LoRaWAN modules or their proposed LoRaWAN modules in the NS-3 simulator [174,175,176,177,178,179,180,181,182,183,184,185,186]. A comparison of NS-3, FLoRa and LoRaSim with a focus on the LoRa/LoRaWAN framework is given in Table 9.

## 4. Methodological Approach

The methodological approach used to analyze and evaluate the selected LoRa/LoRaWAN simulators (i.e., OMNeT++ (FLoRa), LoRaSim and NS-3) in this work is similar to that proposed by the authors in [91]. However, we slightly modified the methodological approach to fit our interests and direction. The methodological approach consists of six steps:

**Step 1. Identify the simulator(s) to evaluate:** The network simulators to be compared and evaluated need to be identified based on criteria to assess the simulators’ various aspects. The network simulators for this purpose were selected based on five criteria:

- The free availability of the simulator for academic and research purposes.
- The active development of new models and protocols by the practitioners and the research community.
- The availability of supporting documentation for the simulators.
- The general purpose of the simulator(s) with respect to the IoT and WSNs applications.
- The growing popularity of the simulators among academics and research communities for the simulation of LoRa/LoRaWAN network.

Based on the above criteria, we selected OMNeT++ (FLoRa), LoRaSim and NS-3 simulators for our analysis. Moreover, for the case of NS-3 LoRaWAN module, we used the NS-3 LoRaWAN Module I for our work. This is because of its excellent documentation and the most preferred module by many research communities.

**Step 2: Establish the experiment setup:** The platform on which the simulators are installed and run should be the same to properly compare and evaluate their performance. For this step, we installed the three simulators on Linux Ubuntu 20.04 LTS platform running on Microsoft Windows 10 version 21H1 with 19043.1466 OS build. The computer specifications are Intel(R) Core(TM) i5-7200U CPU @ 2.50GHz 2.71 GHz with 4.00 GB of RAM (2.2 GB of disk allocated for Linux) and a 64-bit operating system x64-based processor.

**Step 3: Defined the performance assessment/metrics:** More precisely, we evaluate the following metrics:

- *Packet Delivery Ratio (PDR)*: This can be defined as the total number of received packets by the network server divided by the total number of packets sent by the end nodes. The PDR can be computed per node or for the whole network. It is one of the well-known performance metrics in the sensor networks literature. For the entire network, this can be computed as shown by Equation (9):
  (9) $$PDR=\frac{\sum \mathrm{Number}\;\mathrm{of}\;\mathrm{packet}\;\mathrm{received}}{\sum \mathrm{Number}\;\mathrm{of}\;\mathrm{packet}\;\mathrm{sent}}$$
- *CPU Utilization*: This refers to the amount of work a Central Processing Unit (CPU) handles. It is used to estimate the system’s performance. Because some tasks require a lot of CPU time while others require less, CPU utilization can vary depending on the type and amount of computing task.
- *Memory Usage*: This is the memory requirement used by an application while the program executes. It is critical to keep track of memory usage to ensure peak performance.
- *Execution Time*: This refers to the end-to-end time to perform one single simulation run, i.e., the interval between the start and the end time of the simulation scenario.
- *Collisions*: With collision, we refer to the phenomenon that occurs when two or more devices or stations attempt to transmit a packet (data) simultaneously, resulting in the possible loss of transmitted data. Note that the concept of collision or how it is detected may vary depending on how the simulator defines the collision criteria.

**Step 4. Design a test scenario:** A test scenario needs to be designed in each simulator to evaluate their performance. For this work, we designed a small-scale IoT scenario with several sensing nodes and some actuators with LoRa communication technology. Test scenarios are defined by parameters that describe a specific use case or test case execution. For our comparison analysis, we simulated the test scenario with the support of the available LoRa frameworks/modules in these simulators. The scenario consists of a single gateway in a two-dimensional space of 100 m × 100 m and a varying number of EDs around the gateway, ranging from 50 to 400 EDs. The EDs are distributed randomly in the simulation area. The gateway, which is connected to one network server, facilitates communication in the network. To generate a realistic data traffic, we configure the EDs to transmit data packets with 51 bytes and a transmission interval of 100 s.

**Step 5. Execute the designed scenario:** The designed scenario is executed to obtain the needed results for the evaluation. Test scenarios often need to be executed multiple times with variations. In this work, the simulation was run several times for a given number of EDs (six times).

**Step 6. Analyze and evaluate the result(s):** The performance analysis of the simulators is measured based on the obtained results. Users can select the most appropriate simulator(s) according to their needs and applications. Table 10 and Table 11 summarize the main simulation parameters and different versions of the simulators used, respectively.

## 5. Analysis and Discussion of Results

**Figure 8:**
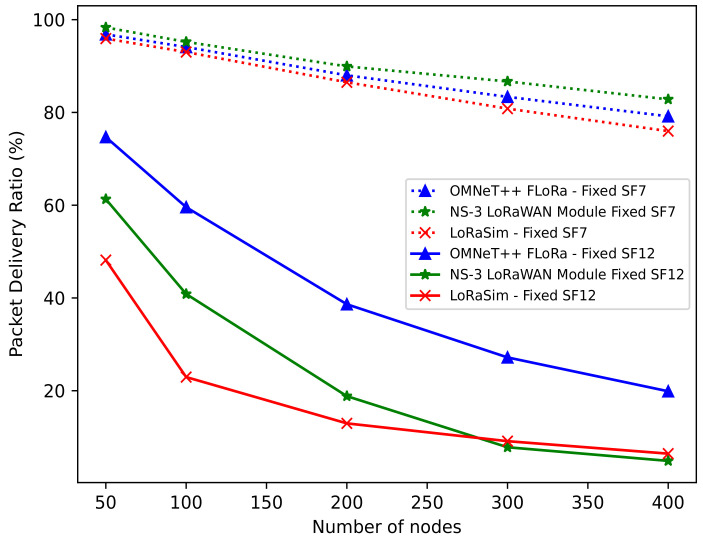
PDR vs. number of nodes.

***PDR***: Figure 8 shows the PDR (%) as a function of the number of nodes for SF = 7 and SF = 12. We set BW = 125 kHz and CR = 4/5 in all configurations. Moreover, the number of nodes ranges from 50 to 400. The results in Figure 8 show that a higher packet success probability is achieved with SF = 7 (dotted lines) due to shorter packet transmission. In contrast, a lower packet success probability is achieved with SF = 12 (solid lines) due to longer packet transmission. Note that shorter packets require more headers than longer packets. Hence, it is not difficult to conclude that a lower SF results in higher PDR, while a higher SF results in lower PDR.

For the simulators, we can see that the NS-3 LoRaWAN module achieved higher PDR with SF = 7, followed by OMNeT++ (FLoRa) and LoRaSim. However, with SF = 12, we observed that for all the simulators, the PDR decreases as the number of nodes increases. In this case, OMNeT++ (FLoRa) shows a much better PDR than both NS-3 LoRaWAN module and LoRaSim. This can be attributed to the fact that OMNeT++ (FLoRa) received more packets than the NS-3 LoRaWAN module and LoRaSim. Therefore, it can be concluded that the NS-3 LoRaWAN module performs better with lower SF while OMNeT++ (FLoRa) performs better with higher SF.

**Figure 9:**
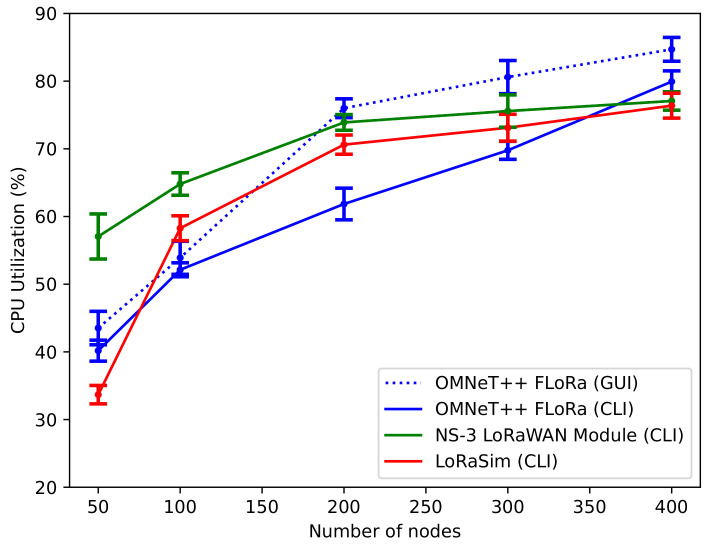
CPU utilization vs. number of nodes.

***CPU utilization***: The CPU utilization (%) for the simulators was measured while varying the number of nodes in the network scenario. Figure 9 shows the average percentage of CPU usage for OMNeT++ (FLoRa), NS-3 LoRaWAN module, and LoRaSim simulators along with the 95% confidence intervals on the plot. Because both NS-3 LoRaWAN module and LoRaSim have only a CLI, we also run the OMNeT++ (FLoRa) simulation using both the CLI and GUI. When the network size is larger, the CPU utilization for the three simulators does not differ much. In particular, the CPU usage at 400 EDs was approximately 76%, 78% and 80% for LoRaSim, NS-3 LoRaWAN module and OMNeT++ (FLoRa), respectively. Thus, compared to OMNeT++ (FLoRa) and NS-3 LoRaWAN module, LoRaSim had the lowest CPU usage percentage at 400 nodes. However, from about 80 to 360 nodes, we observed that OMNeT++ (FLoRa) uses less CPU while the NS-3 LoRaWAN module uses the highest CPU. However, for smaller networks (50–70 nodes), LoRaSim uses the lowest CPU usage. Moreover, the dotted line on the plot depicts the CPU usage for OMNeT++ (FLoRa) when the GUI is utilized. Additionally, we run the simulation using the express mode. We observed a high CPU usage percentage (approximately 85%) when the OMNeT++ (FLoRa) GUI is utilized. This high percentage can be due to the high CPU processing requirements for the GUI.

**Figure 10:**
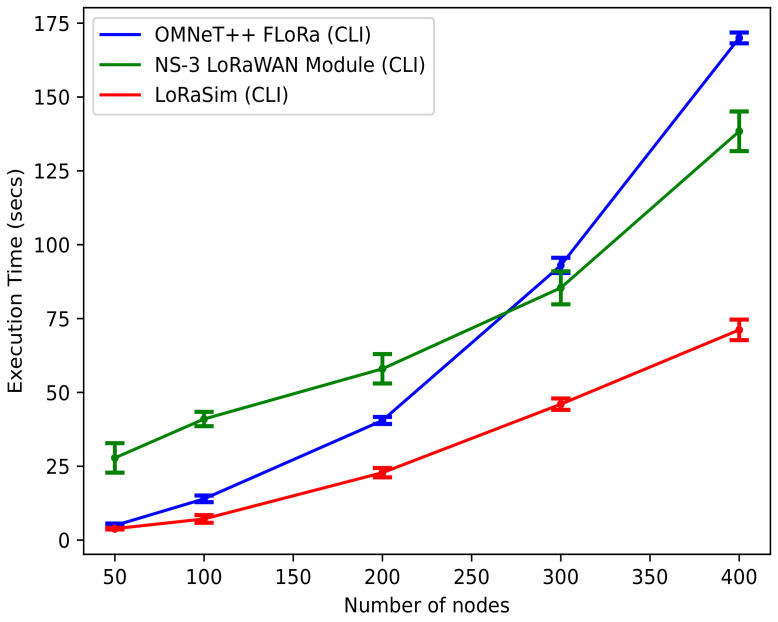
Execution time vs. number of nodes.

***Execution time***: Figure 10 shows the average execution time in seconds versus the number of nodes for the three simulators, along with 95% confidence intervals. We observed that the execution time for the LoRaSim is considerably lower than that of the NS-3 LoRaWAN module and OMNeT++ (FLoRa) simulators. It is also evident that the NS-3 LoRaWAN module has the highest execution time, from 50 to approximately 270 nodes; i.e., the NS-3 LoRaWAN module takes much longer to execute the simulation than OMNeT++ (FLoRa) and LoRaSim. On the other hand, OMNeT++ (FLoRa) appeared to have an average execution time for the scenarios. However, for a large network size (280–400), OMNeT++ (FLoRa) requires more execution time than the NS-3 LoRaWAN module and LoRaSim. In terms of execution time, we can conclude that LoRaSim appears to be the most efficient in this context.

**Figure 11:**
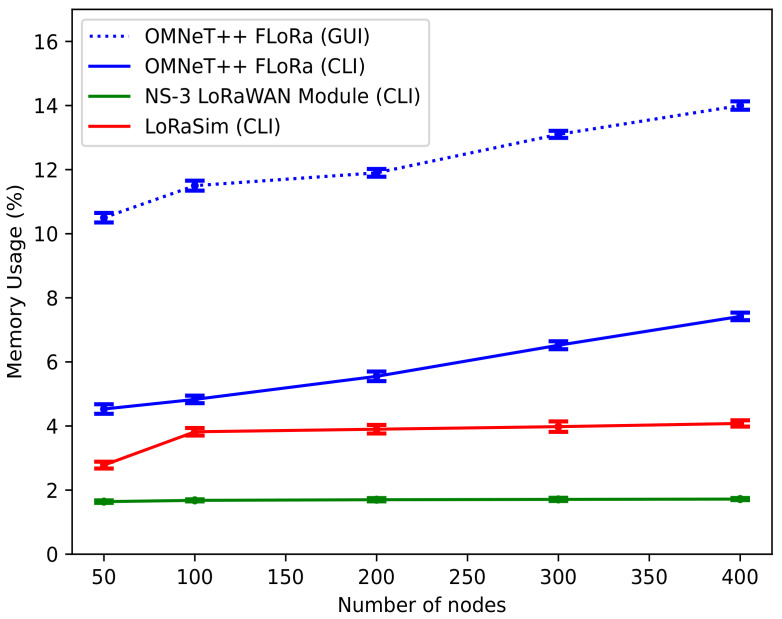
Memory usage vs. number of nodes.

***Memory Usage***: Figure 11 shows the graph of the average memory usage vs. the number of nodes for OMNeT++ (FLoRa), NS-3, and LoRaSim simulators. In the figure, the *x*-axis represents the number of nodes varied from 50 to 400, and the *y*-axis represents the memory usage in percentage (%). Again, a 95% confidence interval is shown in the figure. We observed that as the number of nodes increases, there is somewhat a linear growth in the amount of memory usage for the simulators, with minor differences. The NS-3 LoRaWAN module uses the lowest amount of memory, while OMNeT++ (FLoRa) uses the highest. LoRaSim, on the other hand, appears to use a moderate amount of memory.

Moreover, the memory usage for OMNeT++ (FLoRa) when the GUI is used is shown in the figure with a dotted line. Again, the express mode is used to obtain the memory usage in OMNeT++. We noticed a high percentage of memory usage with the OMNeT++ GUI. Of course, this high percentage of memory consumption can be attributed to the fact that GUI requires relatively more memory as it contains a lot of graphical components. In contrast, CLI does not require more memory consumption or usage. Additionally, every module requires its CPU stack, leading to more significant memory requirements for the simulation program. Overall, the NS-3 LoRaWAN module was found to be the most efficient in this regard.

**Figure 12:**
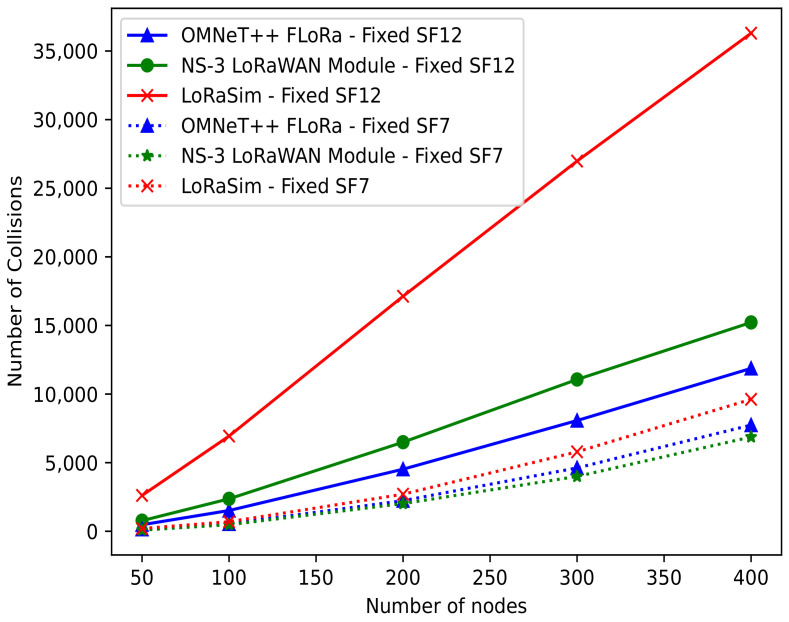
Number of collisions vs. number of nodes.

***Number of collisions***: Figure 12 illustrates the number of collisions occurring in the simulation as a function of the number of nodes. The figure shows that the number of collisions increases linearly when the number of nodes increases. The total number of collisions for a simulation should be minimal to achieve the highest performance. This is because an increased number of collisions lead to network performance degradation. In the figure, we can see that the number of collisions rapidly increases with higher SF. Obviously, with SF = 12, we expect more collisions due to the longer packets. LoRaSim has the highest number of collisions when SF = 12, followed by the NS-3 LoRaWAN module and OMNeT++ (FLoRa). However, with SF = 7, the NS-3 LoRaWAN module has fewer packet collisions. Thus, from a collision point of view, the number of collisions in the NS-3 LoRaWAN module is lower than in the other two simulators with a lower SF value.

## 6. Summary and Conclusions

This paper provides a detailed chronological survey of available IoT and WSNs simulation tools. Specifically, we highlight the most important works from recent studies using a systematic review approach. Next, we present an overview of LoRa/LoRaWAN technologies. We also provide a detailed background on the LoRa/LoRaWAN network, its transmission parameters, classes of its end-devices and available simulation tools. Then, we present a comparative study of three open-source simulation tools/frameworks, namely, NS-3, LoRaSim and OMNeT++ (FLoRa), for the simulation of LoRa/LoRaWAN networks. In each simulator, we equally implemented a simple IoT scenario that used the LoRa communication framework and compared their performance in terms of the Packet Delivery Ratio (PDR), CPU utilization, memory usage, execution time and the number of collisions. The simulation statistics were collected and analyzed at the end of the simulations. Despite the differences in the compared simulators and the obtained results, we would like to acknowledge that each simulator is preferable under different performance measures, depending on the primary research direction and objection.

Finally, many open issues and challenges to developing a more realistic LoRa/LoRaWAN network simulation exist. All the presented LoRa/LoRaWAN simulators have unavailable features in their frameworks that can further be implemented: for example, the incomplete implementation of the LoRaWAN specification as defined by the LoRa Alliance. Moreover, essential features such as interference between partially overlapping channels, confirmed transmission mode, support for classes B and C, duty cycle restrictions, transmission queue, and sophisticated ADR algorithms can be explored. However, because of the free availability (open-source) and the active development of these frameworks by various academic researchers and communities, we expect significant improvement of the available and newly developed simulation tools for LoRaWAN network simulation in the future.

## Figures and Tables

**Table 1:** Contribution of the Reviewed Studies (2011–2021).

Year [Ref.]	Simulators/Emulators	Study Type	Scope of Study
2011 [88]	COOJA, MiXiM, NS-3, OMNeT++, QualNet, Shawn, TOSSIM	Evaluation	Overview, evaluation environment, evaluation approaches and requirements, comparative study of wireless link properties (case study) and comparison table in terms of the simulation model
2011 [52]	AKAROA, GloMoSim, GTNetS, NetSim, NS-2, OMNeT++, OPNET, P2PRealm, QualNet, Shunra VE	Review	Review, classification, comparison, methodologies, techniques and comparison table
2011 [19]	ATEMU, Avrora, EmStar, J-Sim, NS-2, OMNeT++, TOSSIM	Survey	Overview, merits, limitations and comparison table
2011 [55]	GloMoSim, GTSNetS, NS-2, OMNeT++, OPNET, SENSE, TOSSIM	Review	State of-the-art, features, limitations and comparison table
2011 [53]	Castalia, J-Sim, Mixim, NRL, NS-2, OMNeT++, PAWiS, SENSE, SenSim, SensorSim, TOSPIE2	Review	Overview, state-of-art, features and requirements
2011 [63]	NS-2, OPNET, QualNet	Comparative study	Relevance of WSN simulators compared to the IEEE 802.15.4 standard Testbed
2011 [64]	Avrora, Castalia, Cooja, EmStar, GloMoSim, J-Sim, (J)Prowler, NS-2, SENS, SENSE, Shawn, TOSSIM, UWSim, VisualSense	Comparison	Overview, environment, features, simulation/ programming language, limitations and comparison table
2011 [65]	Castalia, MiXiM, TOSSIM, WSNet	Comparison	Examine realistic models topology, energy consumption model, antenna setting, MAC, noise and radio propagation of the simulators/emulators
2012 [20]	AlgoSenSim, Atarraya, ATEMU, Avrora, COOJA, EmSim, Sensor Network Package, Freemote, J-Sim, MSPsim, NetTopo, NS-2 based (NRL Sensorsim, RTNS, Mannasim), OMNeT++ based (PAWiS, MiXiM, SENSIM, NesCT, Castalia), Prowler, Ptolemy II based (VisualSense, Viptos), SENS, SENSE, Sensor Security Simulator (S3), Shawn, TOSSIM, SIDnet-SWANS, TRMSim-WSN, VMNet, Sinalgo, Wireless Sensor Network Localization Simulator, Wireless Sensor Network Simulator, WSim, WSN-Sim, WSNet, WsnSimPy	Survey	Overview, classification, features, applications and comparison table
2012 [21]	J-SIM, NetSim, NS-2, OMNET++, OPNET, NS-3, QualNet, REAL	Survey	Overview, features, advantages and disadvantages
2012 [66]	GloMoSim, J-SIM, NS-2, OMNeT++, OPNET, QualNet	Comparison	Overview, performance comparison, and comparison table
2012 [67]	ATEMU, Avrora, Castalia, J-Sim, NS-2, OMNeT++, OPNET, TOSSIM	Comparison	Overview, merits, limitations and comparison table
2012 [22]	ATEMU, AVRORA, Castalia, (J)Prowler, SENSE	Survey	Brief overview
2012 [23]	Dingo, EmStar, GloMoSim, J-Sim, NS-3, OPNET, QualNet, SENS, SensorSim, Shawn, TOSSF, TOSSIM	Survey	Overview, modeling, methodologies and comparison table
2012 [68]	NS-2, NS-3	Comparison	Overview, features, differences, advantages and disadvantages
2012 [69]	MATSNL, NS-2, OMNeT++, NS-3, PowerTOSSIM, PowerTOSSIM-z	Comparison	Features, performance, reliability, energy consumption, techniques and comparison table
2012 [24]	Glomosim, J-Sim, NS-2, NS-3, OMNeT++	Survey	Overview, features, advantages, disadvantages, future work, limitations and comparison table
2013 [70]	GloMoSiM, NS-2, NS-3, OMNET++	Comparison	Performance comparison
2013 [25]	COOJA, GloMoSim, J-Sim, (J)Prowler, NS-2, OMNeT++ based (Castalia), SENS, SENSE, Shawn, TOSSIM, UWSim, VisualSense	Survey	Overview, classification, features, scalability, effectiveness, limitations and comparison table
2013 [26]	ATEMU, Avrora, J-Sim, NS-2, OMNeT++, Sense, Sensorsim, TOSSIM	Survey	Comprehensive overview and energy/power consumption
2013 [71]	Castalia, J-Sim, TOSSIM, NS-2, QualNet, NS-3	Comparison	Overview, limitation, model, merits and demerits
2013 [27]	J-Sim, NS-2, OMNeT++, NS-3	Comprehensive Survey	Overview, features, architecture, advantages, disadvantages and comparison table
2013 [28]	Avrora, Castalia, GloMoSim, J-Sim, MiXiM, NS-3, OPNET	Survey	Overview and features
2013 [30]	Dingo, EmStar, GloMoSim, GTSNetS, J-Sim, SensorSim, NS-2, TOSSIM, NS-3, Qualnet, SENS, Shawn, TOSSF, OPNET	Survey	Overview, modeling, simulation methodologies, features, drawbacks and comparison table
2013 [31]	J-SIM, NS-2, TINYOS, NS-3, NetSim, OMNeT++, OPNET, SimPy, QualNet	Survey	Overview, advantages and disadvantages
2013 [32]	ATEMU, EmStar/EmSim/EmTOS, J-Sim, GloMoSim, OMNeT++, NCTUns2.0, NS-2, JiST/SWANS, Prowler/(J)Prowler, Ptolemy II, SENS, SNAP, SSFNet, TOSSIM	Survey	Overview, WSN model, framework choice, simulation software package (general and specific) and comparison table
2014 [56]	ATEMU, Avrora, Castalia, COOJA, Dingo, EmStar, GloMoSim, J-Sim, JiST/SWANS, NS-2, NS-3, OMNeT++, SENS, SENSE, SensorSim Shawn, ShoX, Sidh, WsnSimPy, TOSSF, TOSSIM, VisualSense	Review	Overview, features, advantages and disadvantages and comparison table
2014 [33]	GloMoSim, NS-2, OMNET++, NS-3	Survey	Characteristics, limitations, availability (site), applications to MANET, advantages and disadvantages
2014 [72]	NS-2, OMNeT++ (Castalia), NS-3, J-Sim, TOSSIM	Comparison	Overview and performance comparison (CPU utilization, memory usage, computational time period)
2014 [57]	GloMoSim, J-Sim, OPNET, NS-2, OMNET++, NS-3, QualNet	Review	Overview, evaluation methods, routing protocols, advantages and drawbacks, selection criteria, popularity and comparison table
2014 [34]	Castalia, EmPro, EmStar, Freemote Emulator, GloMoSim, MiXiM, MSPSim, NS-3	Survey	Overview, features, types and limitations
2014 [73]	DRMSim, GloMoSim, GrooveNet, J-SIM, NCTUns, NetSim, NS-2, NS-3, OMNeT++, OPNET, QualNet, SSFNet, TOSSIM, TraNS	Comparison	Overview, features, advantages, limitations and comparison table
2014 [94]	AEON, AlgoSenSim, Atarraya, ATEMU, Avrora, Boris, Capricorn, Castalia, CaVi, COOJA, DiSenS, EmStar/Em*, EmTOS, EnergySim, GloMoSim, GTNetS, H-MAS, J-Sim, JiST/SWANS++, JiST/SWANS, (J)Prowler, LecsSim, LSUSensorSimulator, Mannasim, Maple, MOB-TOSSIM, motesim, Mule, NetTopo, NAB, NS-2, OLIMPO, OMNeT++, OPNET, PAWiS, PowerTOSSIMZ, Prowler, Ptolemy, QualNet, SenQ, Sensor security simulator (S3), SENS, SENSE, Sensoria, SensorMaker, SensorSim, Shawn, Sidh, SimGate, SimPy, SimSync, Sinalgo, SmartSim, SIDnet-SWANS, SNAP, SNetSim, SNIPER-WSNim, SNSim, SSFNet, Starsim, TikTak, TOSSF, TOSSIM, TRMSim-WSN, UWSim, VisualSense, Wireless Sensor network localization simulator, WISDOM, WISENES, WiseNet, WSim, WSNet-Worldsens, WSNGE, WSNsim, Xen WSN simulator	Analytical Study	Evaluation criteria, type of simulation, classification/categorization, recent developments, designed or modified and nearby realistic experimental results
2015 [58]	DRMSim, GloMoSim, J-Sim, LabVIEW, Mannasim, MATLAB/Simulink, NCTUns 6.0, NetSim, NetTopo, NRL Sensorsim, NS-2, NS-3, OMNeT++, OPNET, PiccSIM, Prowler, Ptolemy II, QualNet 7.0 and EXata 5, SENS, SENSE, SensorSim, SHAWN, SIDH, SIDnet-SWANS, sQualNet, SSFNet, UWSim, Viptos, Visual Sense, WSim/WorldSen/s/WSNet, WSN Localization	Review	Comprehensive review, architecture, features, interface/GUI, and comparison table
2015 [35]	J-Sim, NetSim, NS-2, OPNET, NS-3, QualNet, OMNeT++	Survey	Overview
2015 [35]	J-Sim, NetSim, NS-2, OPNET, NS-3, QualNet, OMNeT++	Survey	Overview
2015 [95]	ATEMU, AVRORA, Castalia, Emsim, Free Emulator, J-SIM, MPSim, NS-2, QualNet, OMNeT++, Prowler, NS-3, TOSSIM, WSim, WSN Localization Simulator	Qualitative analysis	Overview, classification, features, limitation, pros and cons, and comparison table
2016 [54]	ATEMU, Avrora, Castalia, EmStar, GloMoSim, J-Sim, MiXiM, MSPsim, NesCT, NRL SensorSim, NS-2, NS-3, OMNeT++, OPNET, PAWiS, Prowler/(J)Prowler, SENS, SENSE, SenSim, SensorSim, Shawn, SUNSHINE, TOSSIM	Review	Overview, features, implementation, usage (general networking or for WSNs), techniques, structure and short comparison table
2016 [74]	Avrora, Castalia, COOJA/MSPSim, DANSE, MiXiM, NetTopo, NS-2, NS-3, PASES, PAWiS, Sense, TOSSIM, VIPTOS, WSNet	Comparative study	Overview, categorization, different mainstream simulation environments and comparison table
2016 [75]	Atarraya, MATLAB/Simulink, NS-2, OMNeT++, PiccSIM, Prowler, TrueTime	Comparison	Analyzed and compared various simulation frameworks and comparison table
2016 [36]	Aqua-glomo, Aqua-netmate, Aqua-Sim, Aqua-tools, AUVNetSim, Desert, NS-2, NS-3, OPNET, QualNet, UNSET, USNet, UWSim, WOSS	Survey	Overview, Underwater Sensor Network (UWSN), features, pre-requirements and comparison table
2016 [76]	Castalia, NS-3, TOSSIM	Comparison	Overview, features, power consumption and comparison analysis
2016 [77]	NS-2, NS-3	Comparison	Overview, features, architecture, merits, demerits, models and comparison table
2016 [37]	CNET, Dingo, EmStar, GloMoSim, GTSNetS, J-Sim, TOSSIM, NS-2, OPNET, SENS, SensorSim, Shawn, NS-3, TOSSF, TRMSim, Qualnet	Comprehensive survey	Overview, features, limitations, methodology, test-beds, hardware platforms and comparison table
2016 [92]	Castalia, MiXiM, PASES, WSNet, COOJA	Case study	Routing behavior, protocols, models and accuracy performance
2017 [38]	J-Sim, MATLAB, NS-2, NS-3, OMNeT++, OPNET, QualNet	Survey	Taxonomy on simulation, overview, features, limitations and comparison table
2017 [39]	NS-2, OMNeT++, OPNET Modeler	Survey	Overview, performance analysis and comparison table
2017 [59]	*Simulators*: Ptolemy II and its derivatives (Ptolemy II, Viptos, VisualSense), NS-2 and its derivatives (NS-2, Mannasim, NRL Sensorsim, RTNS, TRAILS, PiccSIM), NS-3 (NS-3, Symphony), OMNeT++ and its derivatives (OMNeT++, SENSIM, LSU SensorSimulator, Castalia, SolarCastalia, MiXiM, NesCT, PAWiS), GloMoSim and its derivatives (GloMoSim, QualNet, SenQ), Worldsens and its derivative (Worldsens, WSNet), Other general-purpose simulators (AlgoSenSim, NetTopo, SENSE, JiST/SWANS, Sinalgo, SimPy, MSPSim, COOJA, J-Sim, NetSim, OPNET, SSFNeT, NCTUns, SystemC, Wireshark, MATLAB SIMULINK, LabVIEW), Specific-purpose simulators (Atarraya, Cell-DEVS), Agent-based simulators (ABMQ, MASON, RepastSNS, NetLOGO, SXCS), Ubiquitous computing simulators (4UbiWise, UbikSim, TATUS), Underwater simulators (UWSim, SUNSET, SUNRISE, DESERT, RECORDS, Aqua-Net, SeaLinx, Aqua-Net Mate, Aqua-Lab, Aqua-Sim, Aqua-Tune, Aqua-GloMo, Aquatools, UANT, WOSS, AUWCN, SAMON, UsNeT), specific-purpose simulators (SIDnet-SWANS, Wireless Sensor Network Localization Simulator, Sensor Security Simulator (S3), Prowler/(J)Prowler, Shawn, TRMSim-WSN, WSNimPy, SENS, IFAS, Sidh, SenSor, Dingo, SNAP, GTSNetS, IDEA1, WiseNet, SimGate, SimSync, SensorMaker, OLIMPO, WISENES, DiSenS, Sensoria, Capricorn, WISDOM, H-MAS, TikTak, SnSim, SNIPER-WSNim, WSNGE, ShoX, PASENS, CaVi, Glonemo, Maestro, CupCarbon, TimSim, JSensor) *Emulators:* TOSSIM and its derivatives (TOSSIM, PowerTOSSIM z, TOSSF, TYTHON, Mule), Avrora and its derivative (Avrora, AEON), Other emulators (ATEMU, EmPro, OCTAVEX, SensEH, HarvWSNet, UbiSec & Sens, Emuli, MEADOWS, Freemote Emulator, VMNet, WSim, EmStar, WiEmu, WiSeREmulator, SUNSHINE, CORE)	Review	Overview, features, evaluation techniques, environments, requirements, operating systems, limitations, frameworks, performance comparison and comparison table
2017 [78]	ATEMU, Avrora, Castalia, COOJA, Dingo, EmStar, GlomoSim, J-Sim, OMNeT++, JiST/SWANS, NS-2, SENS, SENSE, SensorSim, NS-3, Shawn, ShoX, Sidh, TOSSF, TOSSIM, VisualSense, WsnSimPy	Comparative study	Overview, characteristics, modeling energy consumption, modeling mobility, scalability, extensibility and comparison table
2017 [89]	AEON, ATEMU, Avrora, Castalia, COOJA, EmStar, EnergySim, GloMoSim, IDEA1, J-Sim, NS-2, OMNeT++, OPNET, PAWiS, PowerTOSSIM, Prowler, Ptolemy, QualNet, SENSE, Sensim, SensorSim, Shawn, STORM, TOSSIM, UWSim	Evaluation	Overview, energy-aware scheme, features, advantages, limitations, classification method, power consumption model and comparison table
2017 [40]	Avrora, Castalia, Contiki, Prowler, Riot, Shawn, Shox, TinyOS, TRMSim-WSN	Survey	Overview, features, software evaluation and comparison table
2017 [41]	ATEMU, Avrora, Castalia, COOJA, EmStar, J-Sim, NS-2, OMNeT++, SENS, TOSSIM	Survey	Overview, features, advantage, disadvantages, limitations and comparison table
2017 [60]	Castalia, Cupcarbon, J-Sim, NS-2, TOSSIM, OMNeT++, NS-3	Review	Overview, state of art, IoT applications, architectures, simulation tools in IoT, advantages, disadvantages and comparison tables
2017 [79]	NS-2, OMNeT++	Comparison	Brief overview, advantage, limitation and performance comparison
2018 [42]	GloMoSim, NS-3, J-Sim, NetSim, NS-2, OMNeT++, OPNET, JiST/SWANS, QualNet	Survey	Overview, features, protocols, merits, demerits and comparison tables
2018 [90]	CupCarbon, NC-Tuns, NS-2, NS-3, OMNeT++, OPNET Modeler/ Riverbed Modeler, TOSSIM	Evaluation	Overview, features, routing algorithm (modified Dijkstra algorithm) and comparison tables
2018 [43]	NetSim, QualNet, NS-2, OMNeT++, OPNET, NS-3, REAL	Survey	Overview, features, advantages, disadvantages, backend environment, supporting operating system, and minimum hardware requirement
2018 [80]	Avrora, EmStar, J-Sim, NS-2, NS-3, NS4, OMNeT++, QualNet, SENS, TOSSIM	Comparison	Overview, features, limitation, and comparison table
2018 [44]	J-Sim, MATLAB, NS-2, OPNET, QualNet, TOSSIM	Survey	Overview, selection criteria, merits and demerits
2019 [61]	ATEMU, Avrora, Castalia, Cooja, Emsim, Emstar, Freemote, GloMoSim, J-Sim, Mannasim, MSPSim, NS-2, NS-3, OMNeT++, OPNET, Prowler, QualNet, TOSSIM, VMNET	Review	Overview, features, necessity and limitation of testbeds and comparison table
2019 [45]	MATLAB / Simulink, NS-2, NS-3, Prowler	Survey	Overview
2019 [81]	AVRORA, CloudSim, GloMoSim, GNS3, J-Sim, NetSim, NS-2, OPNET Modeler, NS-3, OptSim, Packet tracer, OMNeT++, QualNet, REAL	Comparative study	Overview, features, benefits, disadvantages, limitations and comparison tables
2019 [46]	GloMosim, J-Sim, OPNET, NS-2, OMNeT++, Qualnet	Survey	Overview, features, recent developments and comparison table
2020 [82]	Avrora, NS-2, TOSSIM, OMNeT++, NS-3	Comparative study	Implementation and evaluation process, different testbeds, features, limitations and comparison table
2020 [18]	NetSim, NS-2, QualNet, OMNeT++, NS-3, SWANS	Review	Focus on NS-3 (popularity and flexibility) and comparison table
2020 [91]	NS-2, OMNeT++, TOSSIM	Evaluation	Overview, methodology, application, energy model, performance comparison (CPU consumption, memory usage, execution time, scalability) and comparison table
2020 [62]	COOJA, J-Sim, LabView, MATLAB/Simulink, Mixim or Castlia, NetSim, NS-2, NS-3, OMNeT++, OPNET, TOSSIM, QualNet	Comprehensive review	Experimental analysis, modeling, estimation, interference avoidance, merits, demerits and comparison table
2020 [83]	GloMoSim, MATLAB/Simulink, NetSim, NS-2, TOSSIM, NS-3, SENSE, OMNeT++, OPNET, QualNet	Comparative study	Overview, classification, methodology, Adhoc on Demand Vector Protocol (AODV), clustering protocol, simulation run-time comparison, merits, shortcomings and comparison table
2020 [47]	MATLAB, NetSim, NS-2, OMNeT++, NS-3	Survey	Overview, coverage techniques, comparisons, classification of coverage and practical challenges performance metrics
2020 [84]	ATEMU, EmStar, J-Sim, NS-2, OMNeT++, TOSSIM	Comparison	Overview, advantages and disadvantages and comparison table
2020 [93]	GNS3, MATLAB, NS-2, NS-3, OMNET++, OPNET IT Guru	Case study	Overview, features, evaluation indicators, measurement and valuation levels, and comparison table
2020 [85]	MATLAB/Simulink, NS-2, OPNET, NS-3, OMNeT++	Comparison	Brief description, network simulation methods, classification, time-sensitive Networking (TSN), comparative analysis
2020 [48]	NS-2, TOSSIM, OMNeT++	Survey	Brief overview, mechanism, transmission technologies, challenges, applications of WSN
2020 [49]	cnet, Dingo, EmStar, GloMoSim, J-Sim, NS-2, QualNet, GTSNetS, OPNET, SENS, SensorSim, NS-3, SensorSim-II, TOSSIM, TRMSim-WSN	Survey	Brief review and feasibility analysis
2021 [50]	J-Sim, MATLAB, NetSim, NS-2, NS-3, OMNeT++, OPNET, QualNet	Survey	Short description, different experimental platforms, architecture, features, limitations and comparison table
2021 [51]	CORE, Komondor, Mininet-WiFi, NS-3, OMNeT++/INET, Packet Tracer	Survey	Overview and recommended usage (in terms of mobility, handover, configuration of network devices, wireless packet simulation, signal range, WEP, WPA, 4-way handshake data exchange (RTS/CTS/Data/Ack) and interference)
2021 [86]	MATLAB, NS-2, NetSim, OMNeT++, NS-3	Comparison	Overview, statistical analysis and comparison with respect to Wake-up Receivers
2021 [87]	GloMoSim, J-Sim, JiST/SWANS, MATLAB/Simulink, NetSim, NS-2, QualNet, OMNeT++, OPNET, NS-3	Comparative Study	Reviews on areas of strength, operating system, supported ad hoc technologies, degree of usability and comparison table

**Table 2:** Comparative Performance of the Reviewed Studies (2011–2021).

Ref.	Compared Simulators/ Emulators	Simulation Parameters	Performance Measures	Scenario/Comment
[64]	NS-2, Shawn, TOSSIM	• Simulation Time: 60 s • Number of nodes: 10,000 • X, Y Dimensions: 500 m × 500 m • Rate of sending packet: 250 ms	• Number of nodes vs. Memory usage • Number of nodes vs. Abstraction level • Number of nodes vs. CPU time	Presented a case study of a simple broadcast message application.
[70]	NS-2, OMNeT++, NS-3, GloMoSim	• Simulation Time: 500 s • Number of nodes: 400–2000 • Packet size: 512 kb • X, Y Dimensions: 1000 m× 1000 m • Routing protocol: AODV	• Number of nodes vs. Computational time • Number of nodes vs. CPU utilization • Number of nodes vs. Memory usage	Compared simulators using AODV routing protocol.
[72]	NS-2, TOSSIM, NS-3, J-Sim OMNeT++/ Castalia	• Simulation Time: 500 s • Number of nodes: 400–2000 • Routing protocol: LEACH • X, Y Dimensions: 1000 m× 1000 m • Packet size: 512 kb	• Number of nodes vs. Memory usage • Number of nodes vs. CPU utilization • Number of nodes vs. Computational time	Compared simulators using LEACH routing protocol.
[82]	Avrora, NS-2	• Nodes number: 100 • Com. range: 10,15,20 m • Sensor type: MicaZ • Topology: Static	• Localization accuracy vs Com. range	Implemented QLoP as a case study to study the effectiveness of simulators and testbeds.
[83]	NS-2, OMNeT++, NS-3, MATLAB	• Number of nodes: 50 & 100 • Routing protocol: AODV	• Simulation run-time comparison	Compared simulators using AODV routing protocol.
[91]	TOSSIM, NS2, OMNeT++/INET	• Simulation time: 100 s • Network area: 10 m× 10 m • Sensor nodes: 4, 8, 16… • No. of BC: 1, 2, 4, 8, 16, 32… • Frequency: 1 Hz • Wireless protocols: 802.11b and 802.15.4 • Payload length: 10–90 bytes • Bitrate: 11 Mbps and 250 Kbps	• Time vs. CPU consumption • Number of BCs vs. Memory usage • Number of BCs vs. Execution time • Energy consumption vs. Payload size	**Performance scenarios:** CPU utilization evaluation **Energy consumption** **scenarios** Energy consumption evaluation using 802.11b and 802.15.4.
[92]	Castalia, MiXiM, WSnet, PASES, COOJA	• Simulation time: 3600 s • Network area: 40 m × 60 m • Traffic type/rate (pkt/min): CBR/1 • Network size: 25 • Number of senders: 1, 2, 5, 10, 24 • PHY models: NXP JN5148 • Receiver sensitivity: −85 dBm • Routing protocol: AODV • MAC: IEEE802.15.4 • Data packet size: 64 bytes • RF output power: −3 dBm • Communication channel Model: log-normal shadowing η = 4.0, σ = 20	• Number of nodes vs. Simulation time • Number of nodes vs. Delay • Number of nodes vs. Received packets	**Scenario:** A multi-hop scenario for analyzing the performance of AODV protocol.
[96]	OMNeT++/INET, JiST/SWANS	• VANET scalability: Circular & rectangular road • Time interval: 0.1 s • Number of Vehicles: >5000 • Routing protocol: AODV • Simulation time: 10 s • Execution times: 3 to 10	• Number of vehicles vs. Time for simulations • Number of vehicles vs. Memory consumption	Scalability study focused on VANETs
[97]	OMNeT++, SXCS	• Number of nodes: 10–1000	• Remaining energy vs. Time • Memory usage vs. Number of nodes • Agents proces. time vs. Number of nodes • Remaining energy vs. Time • Packet Loss vs. Number of nodes	Proposed SXCS, a standalone generic simulator for densely distributed embedded systems.

**Table 3:** LoRaWAN Channel Plan based on Deployed Country/Region [123].

Country/Region	Band/Channels (MHz)	Channel Plan
Europe	433.05–434.79, 863–870	EU433, EU863–870
USA, Canada, Mexico	902–928	US902-928
China	779–787, 470–510	CN779–787, CN470–510
Japan	920.6–928.0	AS923-1
Australia	915–928	AS923-1, AU915–928
United Kingdom	863–873, 915–918	EU863–870, AS923-3
India	865–867	IN865–867
South Korea	917–923.5	KR920-923
Russia	864–869.2	RU864–870

**Table 4:** LoRaWAN Device Classes Features and Applications [131,132].

Class Type	Features	Common Applications
**Class A**	• Are often battery-powered sensors • Most energy-efficient communication class • In sleeping mode most of the time • Usually keep long intervals between uplinks • No latency constraint • Uplink message can be sent at any time • Must be supported by all devices	• Environmental monitoring • Location tracking • Fire detection • Animal tracking • Earthquake early detection • Water leakage detection
**Class B**	• An extension of Class A • Lower latency than Class A • Are battery-powered actuators • Do not need to send an uplink to receive a downlink • Shorter battery life than Class A • Synchronized to the network using periodic beacons • Energy-efficient communication class for latency-controlled downlink	• Utility meters • Temperature reporting
**Class C**	• An extension of Class A devices • Are main powered actuators • Consumes higher power than Class A and B • No latency for downlink communication • Usually runs on mains power • Devices which can afford to listen continuously	• Streetlights • Utility meters with cut-off valves/switches

**Table 5:** Effects of SF on data rate, distance, ToA, receiver sensitivity and battery life.

Parameter	Higher SF	Lower SF
Data rate	Lower	Higher
Distance	Travel longer	Travel shorter
ToA	Longer	Shorter
Receiver Sensitivity	Higher	Lower
Battery Life	Shorter	Longer

**Table 6:** Bit rate (kbits/s) for different ranges of SF and BW.

SF	125 kHz	250 kHz	500 kHz
7	5.47	10.94	21.88
8	3.13	6.25	12.50
9	1.76	3.52	7.03
10	0.98	1.95	3.91
11	0.54	1.07	2.15
12	0.29	0.59	1.17

**Table 7:** EU863-870 Data Rates and Maximum Payload Size [123].

Data Rate	SF	BW (kHz)	Bit Rate (bit/s)	Payload Size (Bytes)
0	12	125	250	51
1	11	125	440	51
2	10	125	980	51
3	9	125	1760	115
4	8	125	3125	242
5	7	125	5470	242
6	7	250	11,000	242

**Table 8:** Comparison of LoRa/LoRaWAN Simulators for IoT.

Ref.	Simulation Environment	Type	Language	Target Domain	Operating System	GUI
[137]	LoRaSIM	Discrete-event	Python	Specific	Linux, macOS, Windows	No
[149,150,151,152]	NS-3	Discrete-event	C++, Python	Generic, specific	Linux, Windows	Yes
[153]	OMNeT++(FLoRa)	Discrete-event	C++	Generic, specific	Linux, macOS, Windows	Yes
[154]	CupCarbon	Discrete-event	Java, SenScript	Zigbee, WiFi, LoRa radio	macOS	Yes
[155]	PhySimulator	Discrete-event	MATLAB	Specific	macOS, Windows	No
[156]	LoRaFREE	Discrete-event	Python	Specific	Linux, macOS, Windows	No
[157]	LoRaEnergySim	Discrete-event	Python	Specific	Linux, macOS, Windows	No
[158]	LoRaWANSIM	Discrete-event	MATLAB	Specific	Linux, macOS, Windows	No
[159]	TS-LoRa	Discrete-event	Micropython	Specific	Linux, macOS, Windows	No
[160]	LoRaWAN-SIM	Discrete-event	Perl	Specific	Linux, macOS, Windows	No
[161]	LoRaMACSim	Discrete-event	Python	Specific	Linux, macOS, Windows	No
[162]	LoRa-MAB	Discrete-event	Python	Specific	Linux, macOS, Windows	No
[163]	LoRaWANSim	Discrete-event	Python	Specific	Linux, macOS, Windows	No
[164]	LoRaPlan	Discrete-event	Python	Specific	Linux, Windows	Yes
[165]	AFLoRa	Discrete-event	C++	Specific	Linux, macOS, Windows	Yes

**Table 9:** Comparison of NS-3, FLoRa and LoRaSim Simulation Tools with focus on LoRa/LoRaWAN [121,148,187].

Features	NS-3 LoRaWAN Module	FLoRa Framework	LoRaSim
Base Simulator	NS-3	OMNeT++	Python
Language	C++ and Pyhton	C++	Python
Event	Discrete	Discrete	Discrete
License	Open source	Open source	Open source
Native GUI Support	No	Yes	Only plot
Power Awareness	Yes	Yes	Yes
Low-Power Protocols	Yes	Yes	Yes
Additional Frameworks	Import all libraries online	INET	SimPy, NumPy, matplotlib
Energy Model	Yes	Yes	Yes
ADR Support	Yes	Yes	No
Examples	Yes	Yes	Yes
ACK Support	Yes	Yes	No
Imperfect SF	Yes	No	No
Capture Effect	Yes	Yes	Yes
Device Class	A	A	A
Multi-GW Support	Yes	Yes	Yes
Uplink Confirmed	No	Yes	Yes
Downlink Traffic	Yes	Yes	No
Network Server	Simple	Through IP	Simple
Urban Propagation Models	Yes	Yes	Yes
Popularity in Literature	High	Medium	High
Documentation	Excellent	Good	Good
Community Support	Very Good	Limited	Limited
Energy Consumption	Yes	Yes	Yes
Latest Version /Year	0.3.0/2021	1.0.0/2021	0.2.1/2017

**Table 10:** Simulation Setup Parameters.

Parameters	Values
Simulation Time	10,000 s
X, Y Dimensions	100 m × 100 m
Number of Gateway(s)	1
Packet Size	51 bytes
Network Topology	star-of-stars
Spreading Factor (*SF*)	7 & 12
Number of End-Devices (EDs)	50–400
Bandwidth (*B*)	125 kHz
Time between Packets	100s
Transmission Power (TP)	14 dBm
Carrier Frequency	868 MHz
Code Rate (CR)	4/5

**Table 11:** Simulators versions.

Simulator	Versions
LoRaSim	0.2.1
NS-3/ NS-3 LoRaWAN Module	3.29/0.3.0
OMNeT++/INET/FLoRa	6.0rc1/4.3.7/1.0.0

## Data Availability

Not applicable.
